# Aging-associated Increase of GATA4 levels in Articular Cartilage is Linked to Impaired Regenerative Capacity of Chondrocytes and Osteoarthritis

**DOI:** 10.1101/2025.03.18.643933

**Published:** 2025-03-19

**Authors:** Meagan J. Makarczyk, Yiqian Zhang, Alyssa Aguglia, Olivia Bartholomew, Sophie Hines, Suyash Sinkar, Silvia Liu, Craig Duvall, Hang Lin

## Abstract

Although the causal association between aging and osteoarthritis (OA) has been documented, our understanding of the underlying mechanism remains incomplete. To define the regulatory molecules governing chondrocyte aging, we performed transcriptomic analysis of young and old human chondrocytes from healthy donors. The data predicted that GATA binding protein 4 (GATA4) may play a key role in mediating the difference between young and old chondrocytes. Results from immunostaining and western blot showed significantly higher GATA4 levels in old human or mouse chondrocytes when compared to young cells. Moreover, overexpressing *GATA4* in young chondrocytes remarkably reduced their cartilage-forming capacity *in vitro* and induced the upregulation of proinflammatory cytokines. Conversely, suppressing *GATA4* expression in old chondrocytes, through either siRNA or a small-molecule inhibitor NSC140905, increased the production of aggrecan and collagen type II, and also decreased levels of matrix-degrading enzymes. In OA mice induced by surgical destabilization of the medial meniscus, intraarticular injection of lentiviral vectors carrying mouse *Gata4* resulted in a higher OA severity, synovial inflammation, and pain level when compared to control vectors. Mechanistically, we found that overexpressing GATA4 significantly increased the phosphorylation of SMAD1/5. Our work demonstrates that the aging-associated increase of GATA4 in chondrocytes plays a vital role in OA progression, which may also serve as a target to reduce osteoarthritis in the older population.

## Full Text Availability

The license terms selected by the author(s) for this preprint version do not permit archiving in PMC. The full text is available from the preprint server.

